# Wnt activation and dual SMAD inhibition for induction and maintenance of hindbrain-like neural stem cell from hiPSCs

**DOI:** 10.1016/j.crmeth.2026.101372

**Published:** 2026-03-30

**Authors:** Ziadoon Al-Akashi, Denise Zujur, Nicholas Boyd-Gibbins, Nathalie Eileen Wiguna, Masato Nakagawa, Tetsuhiro Kikuchi, Asuka Morizane, Jun Takahashi, Makoto Ikeya

**Affiliations:** 1 Department of Clinical Application, Center for iPS Cell Research and Application (CiRA), Kyoto University, 53 Kawahara-cho, Shogoin, Sakyo-ku, Kyoto 606-8507, Japan; 2 Department of Life Science Frontiers, Center for iPS Cell Research and Application (CiRA), Kyoto University, 53 Kawahara-cho, Shogoin, Sakyo-ku, Kyoto 606-8507, Japan; 3 Department of Regenerative Medicine, Center for Clinical Research and Innovation, Kobe City Medical Center General Hospital, Hyogo, Japan

**Keywords:** hindbrain, neural stem cell, neuron, pluripotent stem cell

## Abstract

Neurons exhibit region-specific identities corresponding to functional distinctions across different brain areas. Region-restricted neural stem cells (NSCs) have previously been generated from pluripotent stem cells; however, maintaining their regional identity over extended passages remains challenging. Here, we report the generation of hindbrain-like induced NSCs (Hb-LiNSCs) with upregulated hindbrain-specific markers and downregulated forebrain, midbrain, and spinal cord markers under xeno-free and basic fibroblast growth factor (bFGF)-free conditions using three chemicals—CHIR99021 (at a high concentration), a potent activator of the Wnt pathway; A-83-01, a potent inhibitor of the TGF-β/Activin/Nodal pathway; and LDN193189, a potent inhibitor of the bone morphogenetic protein pathway. Hb-LiNSCs maintained their chromosomal integrity, multipotency, and differentiation capacity even after long-term culture for more than 60 weeks. This approach enhances our understanding of neurodevelopmental and neurodegenerative processes in the hindbrain region and paves the way for developing targeted cell-based therapy as well as disease modeling for drug discovery.

## Highlights

• Hindbrain-like NSCs (Hb-LiNSCs) were induced from hiPSCs using small molecules
• The protocol is xeno- and bFGF-free and ensures high reproducibility
• Hb-LiNSCs maintain regional identity and potency for over 60 weeks of culture
• Differentiated neurons from Hb-LiNSCs show functional network activity *in vitro*

## Motivation

The extraction and expansion of human hindbrain stem cells from human embryos have been reported; however, such methods do not allow cell extraction from patients because of the critical anatomical location and associated ethical constraints. Therefore, we sought to induce human iPSCs into hindbrain-like neural stem cells under minimal conditions that enable scalability and reproducibility.

## Graphical abstract

**Figure:**
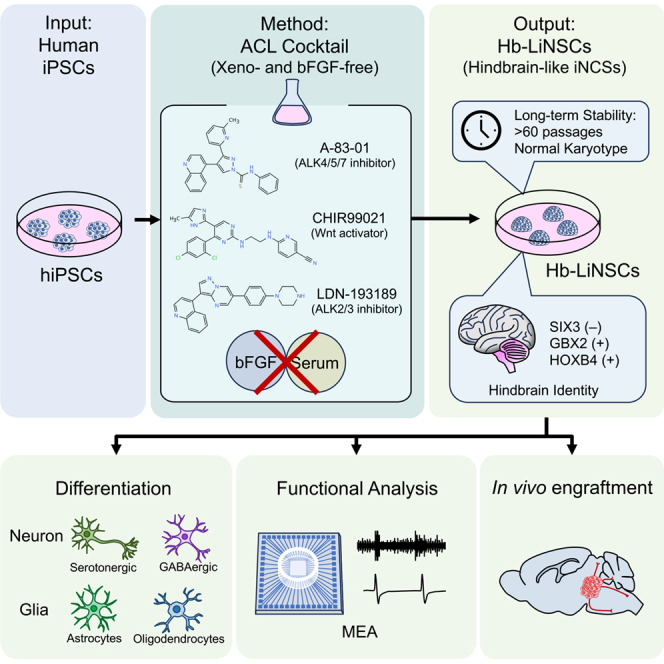

## Teaser

Al-Akashi et al. develop a robust and simplified protocol to generate hindbrain-specific neural stem cells from human iPSCs using a defined small-molecule combination. These cells maintain their regional identity and functional potential for over a year, providing a scalable tool for studying brain development, disease modeling, and potential cell therapies.

## Introduction

Neural stem cells (NSCs) are multipotent cells that arise during development and reside along the neural tube. These cells proliferate and differentiate into the various cell types that form the central nervous system regions during embryological development. NSCs from different regions of the neural tube express specific regional markers.^1^ Since the first report on NSCs in the dentate gyrus of adult rat brains was published over five decades ago, significant progress has been made in isolating NSCs from various brain regions.^2^ Primary and induced NSCs (iNSCs) are invaluable for studying neurogenesis and modeling neurological disorders.

Cells isolated from the hindbrain of aborted human early embryos were reported to maintain their regional specification and neurogenic proliferative capacity even after long-term culture.^3^ Although such an approach provides sustainable, healthy donor-derived hindbrain NSCs, isolating patient-derived hindbrain NSCs from fully developed humans is prone to a great risk of damaging neurons passing through the brainstem that innervate vital organs. Furthermore, the use of human embryos raises ethical concerns.

The use of human-induced pluripotent stem cells (hiPSCs) could overcome the limitations associated with NSC isolation from adult patients and human embryos. hiPSCs provide a scalable source for virtually any cells in the human body, and the ability to obtain hiPSCs from multiple somatic tissue sources renders them useful for disease modeling and regenerative medicine.^4^ The induction of NSCs from several clones of hiPSCs derived from different tissues has been evaluated in previous studies. Dual SMAD inhibition was used to induce mid/hindbrain type NSCs regardless of their somatic origin.^5^ Moreover, activation of WNT signaling was shown to control the rostral-caudal neural axis in a dose-dependent manner using a microfluidic culture device controlling a highly potent and selective GSK inhibitor (CHIR99021), and the development of neural tube expressing the forebrain, midbrain, and hindbrain markers could be modeled using this system depending on the CHIR99021 concentration.^6^

In this study, we aimed to generate iNSCs from hiPSCs having regional specification restricted to the hindbrain (hindbrain-like iNSCs [Hb-LiNSCs]) by upregulating hindbrain gene expression and downregulating forebrain and spinal gene expression under minimal growth factor conditions. To achieve this, we performed a mini-screening and identified high concentration of CHIR99021, along with LDN193189, an inhibitor of bone morphogenetic protein (BMP) type I receptors ALK2/ALK3, and A-83-01, a potent inhibitor of the TGF-β/Activin/Nodal pathway that inhibits ALK5, ALK4, and ALK7, without the use of basic fibroblast growth factor (bFGF) nor serum. Using these minimum induction conditions, we could maintain Hb-LiNSCs for up to 60 weeks in cell culture. We characterized these Hb-LiNSCs during the early (5–20 weeks), mid (25–40 weeks), and late (45–60 weeks) passages and confirmed their long-term maintenance in culture.

## Results

### Induction of Hb-LiNSCs

**Figure 1:**
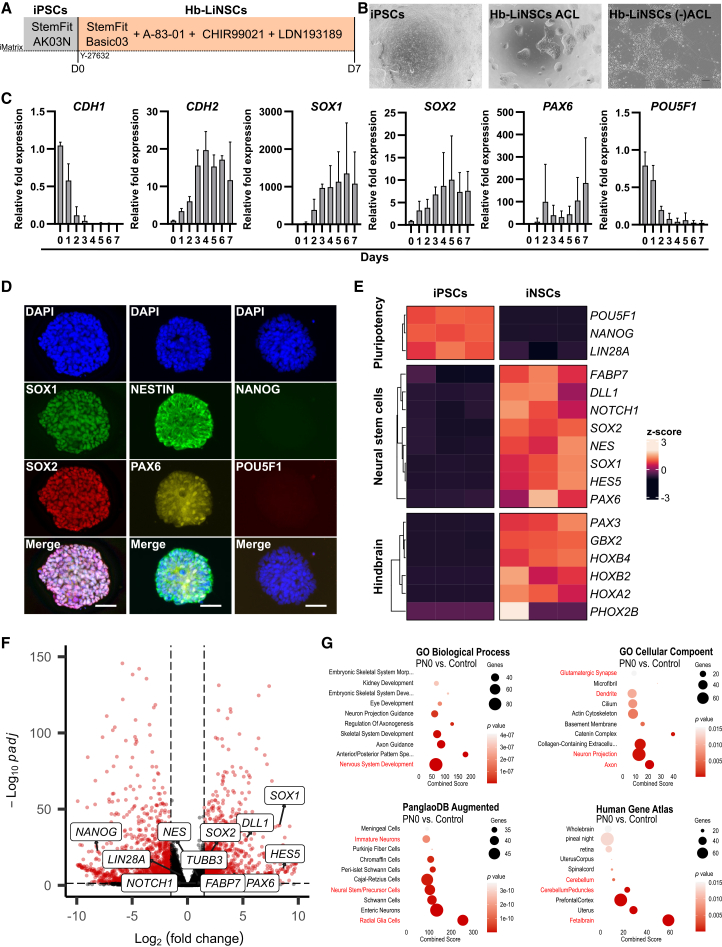
Induction of Hb-LiNSCs (A) Schematic of the method used to induce human induced pluripotent stem cells (iPSCs) into Hb-LiNSCs. (B) Phase contrast images of iPSC and Hb-LiNSCs colonies, and differentiated neurons from left to right. Scale bars, 100 μm. Representative images (*n* = 3, *n* represents the number of independent induction experiments). (C) E-cadherin (*CDH1*), N-cadherin (*CDH2*), *SOX1*, *SOX2*, *PAX6*, and *POU5F1* gene expression in iNSCs during the induction phase relative to that on day 0 (iPS cells). Bars indicate means; error bars as SD (*n* = 3, *n* represents the number of independent induction experiments). (D) Immunocytochemical staining for SOX1, SOX2, NESTIN, PAX6, NANOG, POU5F1, and that of nuclei with DAPI in Hb-LiNSCs at day 7 after induction of iPSCs. Scale bars, 50 μm. Representative images (*n* = 3, *n* represents the number of independent induction experiments). (E) Heatmap of selected genes representing pluripotency, NSCs, differentiated neurons, and canonical markers for the hindbrain region, with hierarchical clustering of genes and samples. Three samples of 1231A3 iPS (as control) and three samples of 1231A3 Hb-LiNSCs at PN0 day 7 were used. Normalized gene expression data are represented by the color intensity of the row *Z* scores. (F) Log fold change values (*x* axis) are plotted against significance (negative log_10_ of *p*-adjusted value, *y* axis) for PN0 day 7 Hb-LiNSC samples (three samples) vs. 1231A3 iPS control (three samples). Red color dots indicate significantly differentially expressed genes. Vertical dashed lines indicate the threshold of 1.5 log fold change, and the horizontal dashed lines indicates the significance threshold (*p* = 0.05). (G) Gene enrichment analysis of the significantly (*p* < 0.05) upregulated (log_2_ fold change ≥1.5) genes in the PN0 Day 7 Hb-LiNSCs vs. iPSC control comparison for selected datasets. Dot size indicates the number of genes overlapping with the dataset, color intensity indicates the significance (top 10 terms ordered by *p* values), and the *x* axis indicates the combined score calculated using Enrichr. Relevant terms are highlighted in red.

Hb-LiNSCs were induced from hiPSCs by inhibiting mesodermal and endodermal pathways through the inhibition of the TGF-β/Activin/Nodal pathway and BMPs using A-83-01 and LDN193189, respectively, and adding posterior characteristics by activating Wnt signaling using CHIR99021 (this condition is hereafter referred to as “ACL”). iPSCs (1231A3) were plated on hLaminin-511E8 fragment-coated culture wells in the same medium used for maintaining iPSCs but without bFGF and with ACL, adding Y-27632 on the first day (Figure 1A). During the first week of culture, multiple independent dome-shaped colonies, which were distinct from the typical confluent (flat) iPSC colonies, were formed. These dome-shaped colonies could spontaneously change their morphology, forming neurite-like projections, when ACL was removed from the culture medium (Figure 1B). The morphology of dome-shaped colonies was maintained after cell passage and was similar in the other two different iPSCs lines tested (a clinical-grade HLA-homozygous line HLAKO and an RNA-reprogrammed line SgT5) (Figure S1A).

Within 3 days, the cells exhibited marked downregulation of E-cadherin (*CDH1*), which is primarily expressed in epithelial cells, and upregulation of N-cadherin (*CDH2*), which is characteristic of neurons. Additionally, the cells gradually upregulated the expression of NSC markers, namely *SOX1*, *SOX2*, and *PAX6*, and downregulated the expression of the pluripotency marker *POU5F1* (Figure 1C). After removing any one or two of the ACL small molecules, the colonies were still formed, and the cells survived through the first week (Figure S1B). Even though the ectodermal markers were upregulated under these conditions, the mesodermal and endodermal markers were also upregulated. Therefore, the use of all three ACL molecules was deemed essential (Figure S1C). The gene expression was confirmed to be consistent in iNSC colonies on day 7 of induction using immunocytochemical staining. The expression of NSC markers, but not of pluripotency markers, was detected (Figure 1D).

To further investigate the ACL induction condition, we utilized bulk RNA sequencing (RNA-seq) to examine the gene expression in three clones of the 1231A3-derived iNSCs for samples from day 7 after induction (PN0 D7). The pluripotency markers were downregulated but the known NSC and hindbrain regional markers were upregulated compared with that in the iPSC samples (Figures 1E and 1F). The pathway enrichment analysis of significantly upregulated genes using public datasets revealed significant enrichment of terms relevant to the nervous system development, such as “Nervous System Development” among the gene ontology (GO) biological process terms and “Glutamatergic,” “Dendrite,” “GABAergic,” “Axon,” and “Neuron Projection” among the cellular component datasets, highlighting the formation of progenitors with multiple neuronal subtypes along with the enrichment of “Immature Neurons,” “Neural Stem/Precursor Cells,” and “Radial Glia Cells” in the PanglaoDB Augmented, and “Cerebellum,” “Cerebellum Peduncles,” and “Fetalbrain” in the Human Gene Atlas datasets, indicating the immature stem/precursor with caudal regional identity of these neurons (Figure 1G). These results indicated the potential induction of hiPSCs into a regionally restricted population of neuronal progenitors, especially those exhibiting hindbrain NSC characteristics.

### Long-term maintenance of Hb-LiNSCs

**Figure 2:**
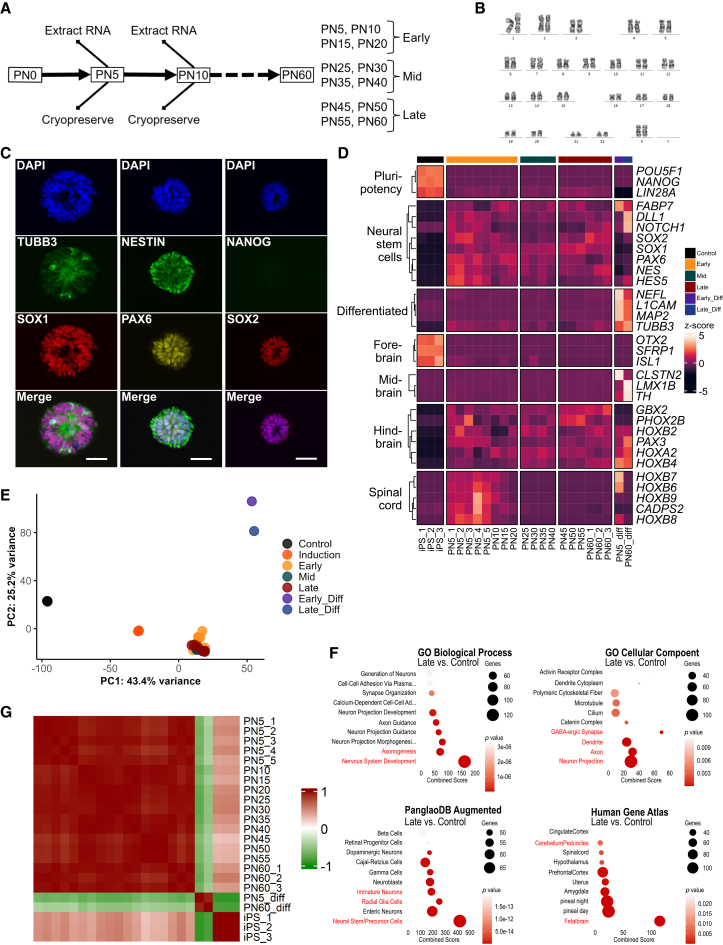
Long-term maintenance of Hb-LiNSCs (A) Schematic of the maintenance plan (on the left). Hb-LiNSCs were passaged every week, and thereafter, at every fifth passage, one clone of cells was cryopreserved, one was used to extract bulk RNA, and one was used for maintaining the culture. The grouping into early, mid, and late samples according to the passage number is shown on the right. (B) Karyotyping of Hb-LiNSCs at PN53 derived from the 1231A3 human iPSC line. (C) Immunocytochemical staining of Hb-LiNSCs for TUBB3, SOX1, NESTIN, PAX6, NANOG, and SOX2, and that of nuclei with DAPI at PN60 (60 weeks) after induction of iPSCs. Scale bars, 50 μm. Representative images (*n* = 3, *n* represents the number of independent induction experiments). (D) Heatmap of selected genes representing pluripotency, NSC, neural differentiation, and canonical markers for different brain regions, with hierarchical clustering of genes. The colored bar on the top indicates the samples. Samples PN5_1 to PN5_3 and PN60_1 to PN60_3 were derived from the 1231A3 iPSC line. Samples PN5_4 and PN5_5 were derived from HLAKO and SgT5 iPSC lines, respectively. “_diff” stands for differentiated neurons. Normalized gene expression data are represented by the color intensity of the row *Z* scores. (E) PCA plots of the first and second (PC1 and PC2) components for the iPSC control are indicated in red, and Hb-LiNSCs and their differentiated cells at early-, mid-, and late-passage numbers are indicated in green, purple, and blue, respectively. (F) Gene enrichment analysis of the significantly (*p* < 0.05) upregulated (log_2_ fold change ≥ 1.5) genes in the late PN group vs. iPSC control comparison for selected datasets. Dot size indicates the number of genes overlapping with the dataset, color intensity indicates the significance (top 10 terms ordered by *p* values), and the *x* axis indicates the combined score calculated using Enrichr. Relevant terms are highlighted in red. (G) Pairwise correlation heatmap showing the relationships among samples. The color intensity in the heatmap indicates the correlation, ranging from 1 (positive correlation) in red through 0 (no correlation) in white to −1 (negative correlation) in green.

To confirm whether Hb-LiNSCs were multipotent progenitor/stem cells, we sought to maintain them in long-term (up to 60 weeks) continuous cell culture, passaged and expanded every week. Every five passages, we cryopreserved subsets of cells and collected cell lysate for RNA analysis and repeated this process until week 60 (Figure 2A). This allowed us to have expandable cell stocks that could be thawed and used for further experiments and to obtain RNA samples at different time points during the long-term maintenance. To confirm that the chromosomal integrity was intact after long-term maintenance, we performed karyotyping and identified no changes even after 53 weeks (benign inversion of chromosome 9 that was observed was preexisting in the 1231A3 iPSC line) (Figures 2B and S2A). To check whether the expression of NSC markers was preserved after the long culture, we performed immunocytochemistry at PN60 and confirmed that the cells uniformly expressed SOX1, SOX2, PAX6, and NESTIN, but not NANOG (Figure 2C).

To gain a comprehensive understanding of the transcriptional changes during long-term culture, we performed bulk RNA sequencing. First, to verify the robustness of our induction protocol across different genetic backgrounds, we compared data from three distinct iPSC lines at the early stage (PN5): biological replicates from the 1231A3 line (PN5_1 to PN5_3), and the other two iPSC line, HLAKO (PN5_4) and SgT5 (PN5_5) (Figure 2D). The high consistency observed across these samples confirmed that our method is not cell-line specific. Furthermore, preliminary analysis of temporal expression patterns in the 1231A3 line suggested that dividing the timeline into approximately 20-passage intervals was appropriate to capture the stabilization of regional identity. Based on this rationale, we then subdivided the samples into three groups of early-, mid-, and late-passage number Hb-LiNSCs based on passage number (time), and compared global gene expression profiles with samples differentiated from early (PN5_diff) and late (PN60_diff) by removing the ACL small molecules and gradually changing the culture medium to neural differentiation medium (NDM). We plotted the results in a heatmap to assess the known genes for pluripotency, NSCs, and differentiated neurons, along with the known markers for the forebrain, midbrain, hindbrain, and spinal cord. Notably, the samples in the early group showed higher expression of the spinal cord and hindbrain markers. Some of these genes, such as *HOXB7* and *HOXB6*, showed a similar pattern of expression even after differentiation (Figure 2D). Despite these differences in expression, Hb-LiNSCs from early, mid, and late samples clustered together and showed slight variance in the principal component analysis (PCA) of the RNA-seq data (Figure 2E). This indicated that the long-term maintenance had a minimum impact on the gene expression profiles. To assess the impact of long-term maintenance on cellular identity, we analyzed the datasets used for Figure 1G, but only used the genes from the late-passage samples (PN45, PN50, PN55, and PN60) that were significantly upregulated compared with their expression in iPSCs. Similar processes and cell identity terms were enriched compared with those in the induction (in Figure 1G) or early samples (Figure S2B). This consistency suggested that the neural stem/precursor state, with fetal hindbrain features, was maintained over time (Figure 2F). Further examination of gene expression patterns in Hb-LiNSCs from passage 5 to passage 60 revealed a high degree of correlation among the expressed genes (Figure 2G). This finding prompted us to further investigate the individual genes exhibiting differential expression across early-, mid-, and late-passage Hb-LiNSCs.

### Transcriptional differences between early- and late-passage Hb-LiNSCs

**Figure 3:**
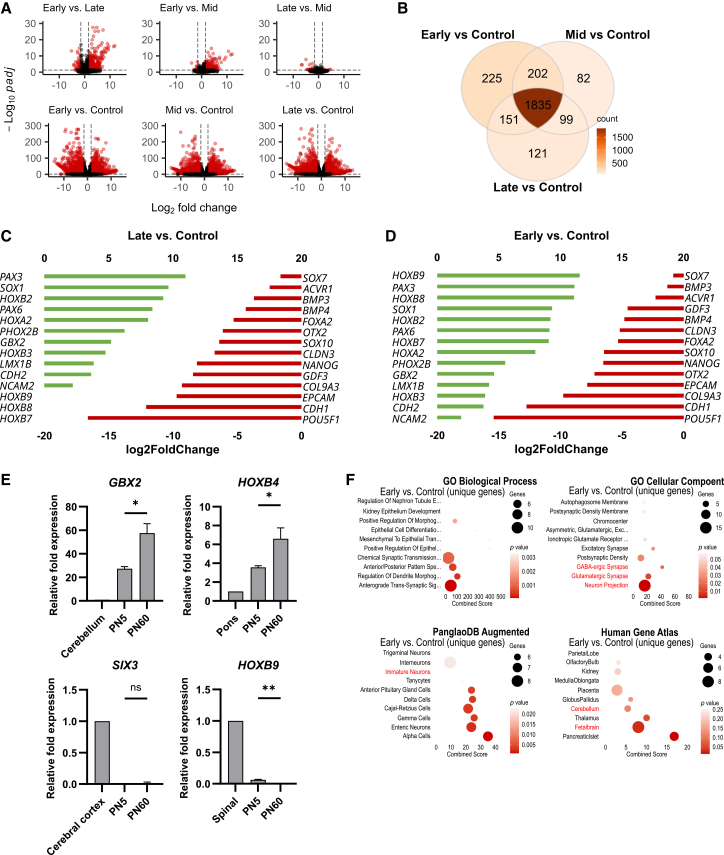
Transcriptional differences between early- and late-passage Hb-LiNSCs (A) Log fold change values (*x* axis) are plotted against significance (negative log_10_ of *p*-adjusted value, *y* axis) for Hb-LiNSCs samples at one phase of maintenance vs. another (top) and for Hb-LiNSCs samples at different phases against the 1231A3 iPSC control (bottom). Red color dots indicate significantly differentially expressed genes (SigDEGs). Vertical dashed lines indicate the threshold of 1.5 log fold change, and the horizontal dashed line indicates the significance threshold (*p* = 0.05). Number of SigDEGs is indicated on top of the plots. (B) Venn diagram of the significantly (*p* < 0.05) upregulated (log_2_ fold change ≥ 1.5) genes in the Hb-LiNSC sample at different phases. Color intensity indicates the count of genes (darker color indicates a higher value). (C) Bar plot of the log fold change of selected genes from the “early vs. control” bulk RNA-seq differential gene expression data. On the left, the list of genes with bars in green indicates upregulated expression (top *x* axis). On the right, the list of genes with bars in red indicates downregulated expression (bottom *x* axis). (D) Bar plot of the log fold change of selected genes from the “late vs. control” bulk RNA-seq differential gene expression data. On the left, the list of genes with bars in green indicates upregulated expression (top *x* axis). On the right, the list of genes with bars in red indicates downregulated expression (bottom *x* axis). (E) Bar plot of RT-qPCR data for *GBX2*, *HOXB4*, *SIX3*, and *HOXB9* in PN5 and PN60 HbL-iNSCs samples (*n* = 3, *n* represents the number of independent induction experiments) relative to human-derived RNA samples from the cerebellum, pons, cerebral cortex, and spinal cord. Bars indicate means; error bars as SD (*n* = 1, *n* represents the number of independent human RNA sample). (F) Gene enrichment analysis of the 427 unique genes in the “early vs. control” comparison for selected datasets. Dot size indicates the number of genes overlapping with the dataset, color intensity indicates the significance (top 10 terms ordered by *p* values), and the *x* axis indicates the combined score calculated using Enrichr. Relevant terms are highlighted in red.

By comparing samples from early-, mid-, and late-passage numbers, we observed a gradual shift toward stabilization (Figure 2D). In the early vs. late comparison, we observed 1,225 significantly differentially expressed genes (SigDEGs), of which 408 were upregulated (≥1.5-fold change); in the early-passage vs. mid-passage sample comparison, we identified 547 SigDEGs, of which 207 were upregulated; whereas in the late-passage vs. mid-passage sample comparison, only 153 SigDEGs were identified, with only 8 being upregulated (Figures 3A and S2C). Similar gradual decrease in the numbers of upregulated genes was observed when we compared early vs. control, mid vs. control, and late vs. control samples (Figure 3B). Although most of the significantly upregulated genes (1,835 genes) were common between early, mid, and late samples, we could still observe the highest number of unique genes (225 genes) in the early vs. control comparison. This indicated that the early sample was more distinct from the mid and late samples. Therefore, we looked at the expression of several canonical genes for the embryological development of germ layers and regional marker genes. A similar level of downregulation of genes related to the mesoderm, endoderm, and pluripotency was noted. *HOXB9* was the gene with the highest expression in our list in the early vs. control comparison, whereas its expression was lost along with that of *HOXB8* and *HOXB7* in the late vs. control comparison (Figures 3C and 3D). *HOX 1–4* genes correspond to the hindbrain regions, whereas *HOX 5–13* correspond to the spinal cord. Furthermore, we examined the expression of *GBX2*, which is a transcription factor in more rostral regions of the hindbrain, and of *HOXB4*, which represents the more caudal regions of the hindbrain along with that of *SIX3*, which is a transcription factor regulating the forebrain, and of *HOXB9* as a marker for the spinal cord.^7^^,^^8^^,^^9^ Significant differences were observed in the expression of the hindbrain markers between samples from early and late passages; however, both showed upregulation relative to that in RNA samples derived from adult healthy human pons and cerebellum (Figure 3E). Although the expression of *SIX3* remained comparatively the same and lower than that in the human-derived sample, *HOXB9* showed a significant difference between early and late passages even though the expression was still markedly lower than that in the human RNA sample from the spinal cord. To further understand the significance of differences in the early samples, we performed a pathway enrichment analysis for the genes that were significantly upregulated and unique to only the early vs. control group comparison (the 225 genes shown in the Venn diagram in Figure 3B). These unique genes showed enrichment of “Anterior/Posterior Pattern Specification” in the GO biological process but less significant enrichment of the other terms, namely “Immature Neurons” in the PanglaoDB Augmented dataset and “Fetalbrain” in the Human Gene Atlas dataset (Figure 3F). Upon direct comparison of the groups against each other, we found the highest number of unique genes in the early vs. late group comparison. We looked at these genes and found that *HOXB6-9* was consistently among the top 15 SigDEGs (Figures S2D–S2F). These findings may indicate that the changes occurring after long-term maintenance are less likely to be related to maturation but more likely to be region-restricted specifications.

### scRNA-seq of early- and late-passage number Hb-LiNSCs reveals distinct cell populations

**Figure 4:**
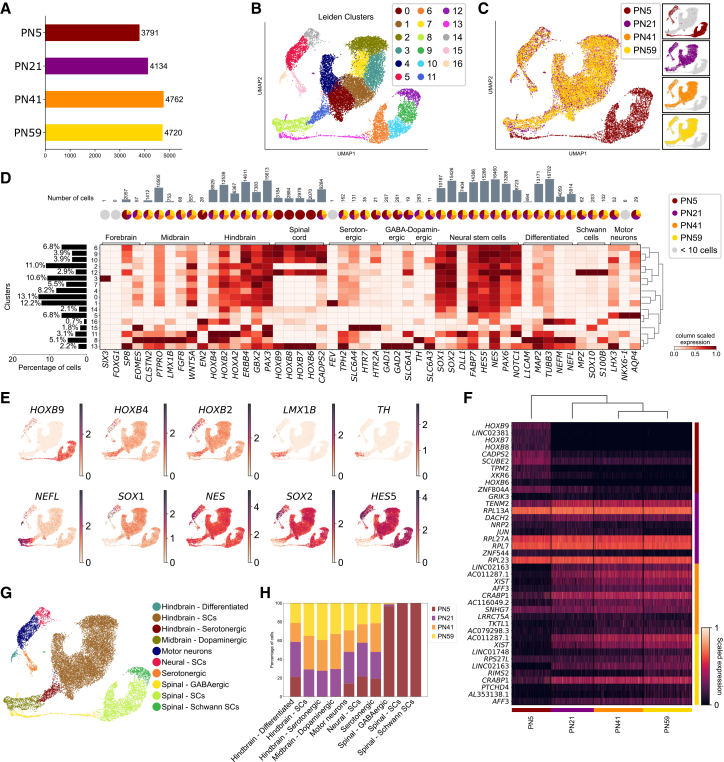
scRNA-seq of early- and late-passage number Hb-LiNSCs reveals distinct cell populations (A) Bar plot showing the number of cells in each sample ordered by passage number. (B) UMAP visualization of scRNA-seq data representing early (PN5), mid-early (PN21), mid-late (PN41), and late (PN59) hindbrain-like NSC (Hb-LiNSC) samples from individual culture wells. Each dot on the plot corresponds to a single cell and is colored based on cluster assignments obtained via Leiden clustering (see “methods” for more details). (C) Left: UMAP plot representing cells color-coded by their respective samples. Right: individual subplots highlighting each sample. (D) Heatmap showing the relative gene expression across clusters identified via Leiden for canonical markers associated with the brain regions, NSCs, and differentiated neurons. Rows are hierarchically clustered. On the left side, the bar plot shows the percentage of cells in each cluster. At the top, the bar plot indicates the number of cells expressing each gene, and the pie chart indicates the proportion of each sample expressing each gene; gray circles denote genes expressed in fewer than 10 cells. (E) Expression of selected genes across the cells on the same UMAP in (A). (F) Heatmap illustrates the expression levels of the top 10 differentially expressed genes across sample groups. Rows represent genes, and columns represent samples, with the color scale indicating standardized expression from low (darker shades) to high (lighter shades). Dendrograms are included for gene and sample clustering. (G) UMAP representation of the same dataset in (A), with each cell color-coded by the specific cell types, determined via the expression of canonical genes. “SCs” refer to stem cells (H) Stacked bar plot showing the distribution of cell types across samples. Each bar represents a cell type, with colored segments indicating the proportion of cells from different samples.

To investigate the differences between early- and late-passage Hb-LiNSCs at a single-cell level, we obtained samples at different time points (PN5, PN21, PN41, and PN59) to represent the early, early-mid, late-mid, and late passages, respectively, and performed scRNA-seq. Different but comparative cell numbers were used in the analysis for each sample (Figure 4A). To determine the heterogeneity within samples, we used the Uniform Manifold Approximation and Projection (UMAP) together with the Leiden clustering algorithm and identified 16 distinct clusters (Figure 4B). We then labeled the cells with their corresponding samples and found that all the clusters were present in all the samples except for clusters 6, 9, 10, 12, and 13, which were represented almost strictly by the PN5 sample (Figure 4C). We examined the expression of canonical marker genes in samples for the forebrain, midbrain, and hindbrain regions of the neural tube along with markers for NSCs and differentiated neurons, such as serotonergic, GABAergic, dopaminergic, and others, which were designated as “differentiated.” The heatmap showed that the genes present in the highest number of cells were those related to the NSCs and hindbrain region, whereas spinal cord markers were strictly present in the PN5 sample (Figure 4D). Clusters 0, 1, 2, 3, 4, and 7 were in proximity and revealed the highest percentages of cells expressing markers for both the hindbrain region and NSCs. Moreover, the expression of *HOXB9* was almost exclusively expressed in the cluster corresponding to the PN5 sample, whereas *HOXB4*, *HOXB2*, *SOX1*, *NES*, *SOX2*, and *HES5* were expressed in almost all the clusters (Figure 4E). Similarly, the heatmap for the expression of the top 10 differentially expressed genes across samples revealed that *HOXB9* was the top differentially expressed gene in cells from PN5, followed by *HOXB7* and *HOXB8* at the third and further position, respectively (Figure 4F). As indicated by the fold difference in expression for the top 25 genes in the PN5 vs. PN59 comparison, many of the *HOX* genes were upregulated (Figure S3A). In the comparison of each sample with PN59, the score was highest for the genes differentially expressed in the PN5 vs. PN59 comparison than in the PN21 or PN41 vs. PN59 comparison, highlighting the major difference being between PN5 and other samples; this difference gradually faded with increasing passage number (Figures S3B and S3C). These results allowed us to re-label the clusters with their indicative cell types, highlighted by the largest cluster belonging to the hindbrain stem cells (Figure 4G). An analysis of the distribution of cell types within the samples showed that cells related to the spinal cord were almost entirely restricted to the PN5 sample (Figure 4H). These results may indicate that, although there is a population of cells expressing both the spinal cord and hindbrain marker genes, this population transitioned over time with long-term maintenance. However, this shift did not affect the number of cells expressing the hindbrain NSC markers, which remained the highest in all the samples (Figure S3D).

### Differentiation and functional network activity

**Figure 5:**
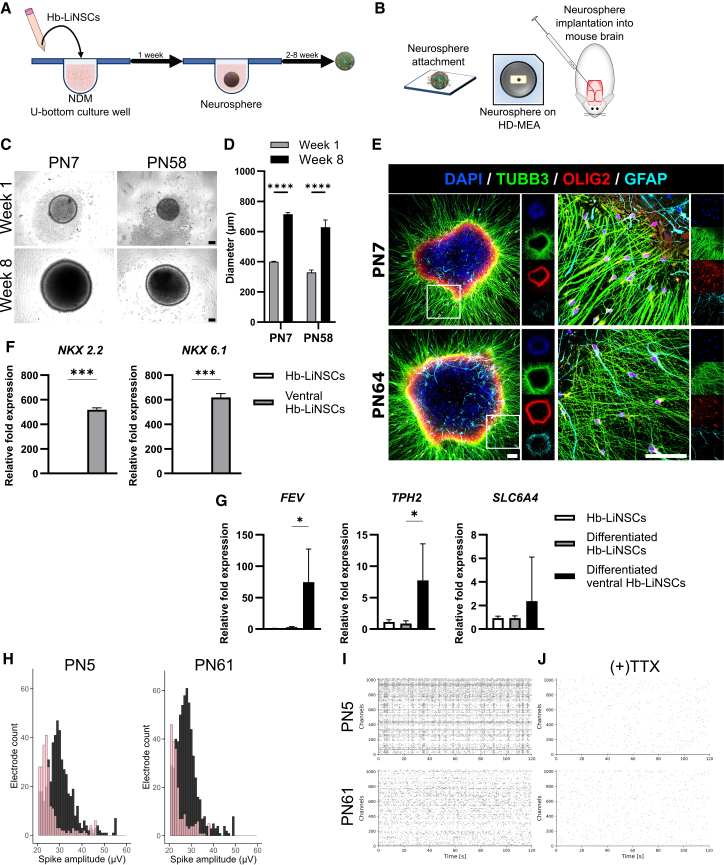
Differentiation and functional network activity (A) Schematic representation of neurosphere formation. (B) The application of neurospheres in attachment to a flat surface, attachment to MEAs, and *in vivo* transplantation. (C) Phase-contrast images of the neurospheres in suspension after 1 week and 8 weeks of seeding. Scale bars, 100 μm. (D) Bar plot for the cross-sectional area of the neurospheres. Bars indicate means; error bars as SD (*n* = 5, *n* represents the number of individual neurospheres). (E) Immunocytochemical staining of the attached neurospheres for TUBB3, OLIG2, and GFAP, and for nuclei with DAPI. Scale bars, 100 μm. Representative images (*n* = 3, *n* represents the number of individual neurospheres). (F) RT-qPCR for gene expression of ventral hindbrain markers (*NKX 2.2* and *NKX 6.1*) in the ventralized Hb-LiNSCs relative to that in the non-ventralized Hb-LiNSCs. Bars indicate means; error bars as SD (*n* = 3, *n* represents the number of individual biological replicates, each consisting of RNA pooled from 5 to 8 neurospheres). (G) Gene expression for serotonergic markers (*FEV*, *TPH2*, and *SLC6A4*) for differentiated neurons relative to that in undifferentiated Hb-LiNSCs. Bars indicate means, with error bars showing the SD (*n* = 3, *n* represents the number of individual biological replicates, each consisting of RNA pooled from 5 to 8 neurospheres). (H) Histogram of the electrode counts for the detected spike amplitudes on the MEAs. The sample (PN5 left plot and PN61 right plot) before tetrodotoxin (TTX) treatment is shown in the dark, and that after TTX treatment is shown in pink. (I) Raster plot of the spike activity detected in the MEA overtime. Each dot represents an action potential (spike) for PN5- (top plot) and PN61-derived (bottom plot) samples. (J) Same samples as in (I) were recorded after adding TTX. Representative plots of 4 recordings of each PN5 and PN61.

Neurospheres are a cluster of cells cultured in a 3-dimensional (3D) manner floating in low-attachment round bottom plates, which are commonly used to assess primary or induced neural cells (Figure 5A). We used the neurosphere method following the removal of the ACL molecules and cultured the Hb-LiNSCs in NDM to differentiate them. We then performed attachment and differentiation analyses, electrophysiological recording, and *in vivo* implantation (Figure 5B). We seeded dissociated cells without any scaffolding material, and these cells formed sphere-like aggregates. These spheres were almost equal in size at initial seeding and exhibited significant growth after several weeks (Figures 5C and 5D). When Hb-LiNSC-derived neurospheres were attached to a flat surface coated with laminin, TUBB3-positive neurite-like projections were observed along with cells migrating outside the sphere edges (Figure 5E). Glial cells, such as astrocytes and oligodendrocytes, positive for GFAP and OLIG2, respectively, were observed. This differentiation capacity was maintained even after the long-term maintenance of Hb-LiNSCs.

iNSCs can be further restricted to the ventral hindbrain region by treating the cells with sonic hedgehog (SHH) recombinant protein and FGF4, resulting in a population of serotonergic progenitor cells expressing *NXK 2.2* and *NKX 6.1* that can differentiate into serotonergic neurons expressing *FEV*, the serotonergic neurons transcription factor, *TPH2*, the tryptophan hydroxylate 2, and *SLC6A4*, a serotonin transporter, which are the key markers for serotonergic neurons found in the central nervous system.^10^ We treated the Hb-LiNSCs with smoothened agonist (SAG) along with FGF4 and obtained ventral Hb-LiNSCs expressing *NXK 2.2* and *NKX 6.1* at significantly higher levels than in the non-ventralized Hb-LiNSCs (Figure 5F). Upon differentiation of the ventral Hb-LiNSCs, the neurons significantly upregulated the expression of *FEV* and *TPH2*, but the difference in expression of *SLC6A4* was not significant when compared to that in non-ventralized Hb-LiNSCs (Figure 5G). This finding highlights the importance and the identity of Hb-LiNSCs as they can be ventralized and differentiated into highly specialized restricted type of neurons present in that region of the brain.

To evaluate the electrophysiological activity of Hb-LiNSC-derived neurospheres, we employed multielectrode arrays (MEAs). After 1 week of sphere formation, followed by 5–6 weeks of maturation and migration of the attached neurospheres to the electrodes, we detected bursts of action potentials (spikes) across multiple electrodes, with amplitudes exceeding 50 μV (Figure 5H). Although neurospheres derived from Hb-LiNSCs at later passages exhibited higher spike amplitudes across more electrodes, the firing rate was greater in those derived from Hb-LiNSCs at earlier passages (Figure S4A). Additionally, repetitive firing was observed from some electrodes within milliseconds, followed by periods of rest and subsequent resumption of firing. Synchronous firing from multiple electrodes suggested the presence of network activity (Figure 5I). Upon adding tetrodotoxin (TTX), a neurotoxin that blocks sodium channels and thereby inhibits the conduction of action potential in neurons,^11^ to the neurosphere culture, spike amplitudes were markedly reduced, and fewer electrodes exhibited activity compared to pre-TTX conditions (pink bars in Figure 5H). The frequency and network activity were also suppressed by TTX (Figures 5J and S4A). These findings indicated that long-term maintenance did not affect the differentiation potential, and Hb-LiNSCs could still generate functional neurons.

### *In vivo* transplantation

**Figure 6:**
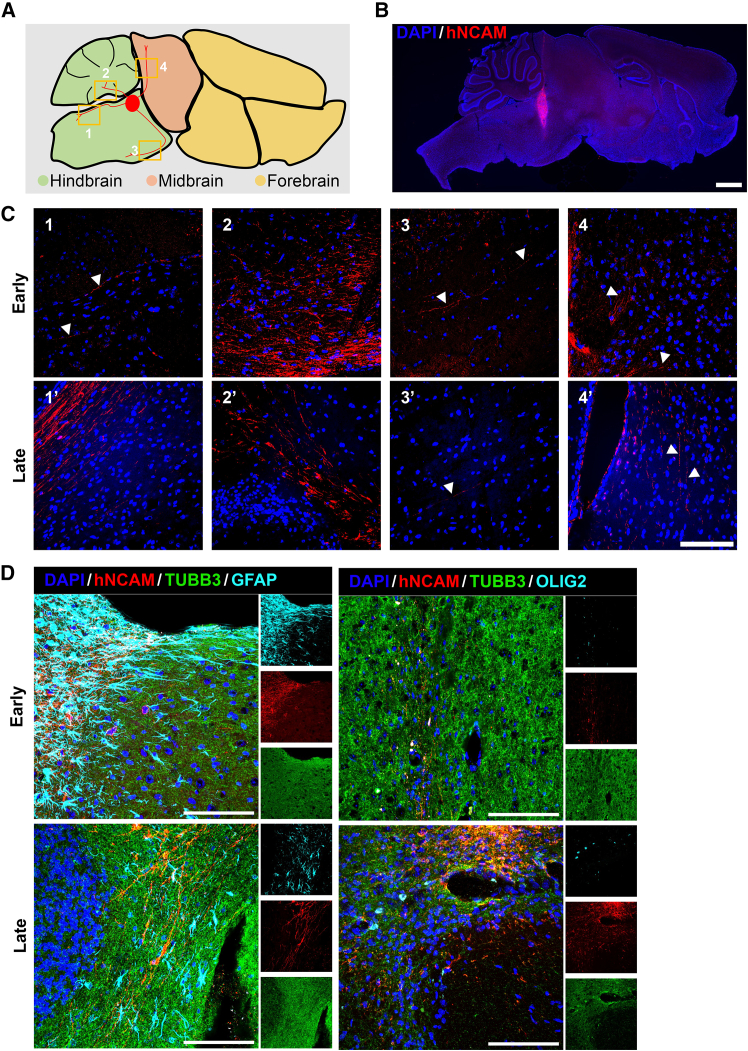
*In vivo* transplantation (A) Schematic of the brain regions showing the transplantation site and the projection of neurite into the regions highlighted by orange squares and numbers. (B) Sagittal section of the mouse brain. (C) Tracking of hNCAM-positive cells along the spinocerebellar tract (1, 1′), cerebellum (2, 2′), corticospinal tract (3, 3′), and in the midbrain (4, 4′). (D) Differentiation of hNCAM-positive cells into TUBB3-, GFAP-, and OLIG2-positive cells in early- and late-passage samples. Representative images (*n* = 3, *n* represents the number of individual mice).

Finally, to investigate the integration and migration of Hb-LiNSCs *in vivo*, we transplanted 1-week-old neurospheres derived from early- and late-passage Hb-LiNSCs into mice brains, interior to the cerebellum and into the pontine region (Figure 6A). After 8 weeks of transplantation, mice were sacrificed, and the integration and migration of the human neural cell adhesion molecule (hNCAM)-positive neural cells was detected using a human-specific NCAM antibody (Figure 6B). hNCAM-positive neural cells were detected in the spinocerebellar tract, cerebellum, corticospinal tract, and midbrain from samples derived from early- and late-passage Hb-LiNSCs (Figure 6C). The transplanted hNCAM-positive neural cells also showed TUBB3-, GFAP-, and OLIG2-positive cells (Figure 6D). These results indicated that the Hb-LiNSC-derived cells can differentiate and extend along with the host neurons, simulating normal physiological development and rendering useful as a tool for disease modeling and potential cell therapy.

## Discussion

We successfully generated Hb-LiNSCs from hiPSCs under minimal induction conditions with small molecules without the addition of bFGF or serum. Specifically, the exclusion of bFGF reduces costs, while the absence of serum eliminates the unknown effects of undefined components, leading to a highly reproducible system. Importantly, compared to the direct differentiation protocols, our Hb-LiNSCs method offers distinct functional advantages. First, it provides unlimited scalability, allowing exponential expansion for over 60 weeks, whereas direct differentiation methods yield a finite number of cells. Second, long-term culture consolidates enhanced regional identity by suppressing forebrain/midbrain markers, resulting in a more homogenous population. Throughout this long-term maintenance, the Hb-LiNSCs retained their chromosomal identity, genetic profile, differentiation potential, and functional activity. Furthermore, our protocol effectively suppressed non-neural lineages during long-term culture. This is likely attributable to the specific actions of the small molecules used: A-83-01 inhibits the TGF-β/Activin/Nodal pathway, which is essential for endodermal differentiation, while LDN193189 inhibits BMP signaling, a key driver of mesodermal specification. Consequently, the continuous presence of these dual SMAD inhibitors creates a selective environment that eliminates mesodermal and endodermal contaminants, thereby ensuring the purity of the neural lineage. Thus, our unique method adds valuable insights to the development of neural regional identity over long periods of stem cell maintenance, with cell passaging.

The regional specification is important in determining cell identity. The forebrain neurons are associated with higher cognitive functions, such as memory and learning; the hindbrain neurons regulate autonomic functions, and the spinal neurons are essential in relaying information from and to the brain. Moreover, pathologies arising from certain populations of neurons have dependencies on the region in question. This highlights the importance of using Hb-LiNSCs in disease modeling. One potential application of Hb-LiNSCs is in the modeling of certain types of malignancies referred to as diffuse interstitial pontine glioma (DIPG). In DIPG, a fatal tumor that affects children, the cells of origin are regionally restricted to the ventral pons.^12^ Another potential application is in the modeling of disorders related to the serotonergic system. Serotonergic neurons are a highly specialized type of neurons that can utilize the neurotransmitter serotonin for their communication and cause disorders, such as major depressive disorder, autism spectrum disorder, Rett syndrome, and others. This potential can be expanded when Hb-LiNSCs from patient-derived iPSCs are established and then combined with MEAs to analyze their network activity, learning rate, and prediction accuracy by integrating neurons into a system that can analyze their spike (action potential) patterns data, such as the MEA-NAP, DishBrain, and BiœmuS.^13^^,^^14^^,^^15^^,^^16^ Our Hb-LiNSCs have become a powerful research tool in this field, although there is a need for further improvement to leverage them for disease modeling and, potentially, drug screening.

We highlighted the differences between early- and late-passage Hb-LiNSCs, focusing on the gene expression evaluated using the sequencing data. We did bulk RNA sequencing of samples at different time points and compared data for those in the early induction phase and those maintained for the long term. Long-term maintenance did not affect maturation, and the terms related to immature/fetal neurons remained similarly enriched in both the early and late stages. scRNA-seq data for samples at different time points of maintenance revealed the largest number of cells (approximately 60%) expressing markers for the hindbrain region and coexpressing NSC markers. The second largest number of cells (approximately 14%) expressed spinal markers and coexpressed stem cell makers. Although the difference between early and late passages could not be fully understood, it is unlikely that maturation is involved; rather, based on the terms enriched for the differentially expressed genes, it is more likely that a higher regional realization occurs (Figure S3E). Cells expressing spinal cord markers were only present in samples from early passages. This finding is not unique to our iNSCs; other researchers have shown that long-term maintenance results in higher expression of the forebrain and midbrain markers, probably due to CHIR99021 inhibiting the FGF targets, resulting in regression of the fate from the spinal cord.^17^ The presence of the spinal cord markers in the early-passage cells did not affect the other characteristics of NSCs with regard to gene expression, freeze/thaw, hindbrain regional markers, differentiation potential, and *in vivo* integration and migration. On the contrary, the electrophysiological activities were slightly different between the early and late passages. The number of electrodes in the MEAs detecting higher spike amplitudes was less for the neurons derived from early-passage cells. In contrast, the frequency was higher for the samples derived from early-passage cells when compared with the late passage cells. The underlying cause for this difference remains unclear, but it could be due to the early-passage cell-derived neurons being regionally more heterogeneous, resulting in different network dynamics when compared with the late passage cell-derived neurons.

### Limitations of the study

While our study establishes a robust method for generating and expanding Hb-LiNSCs, there are limitations that should be addressed in future research. First, we validated the hindbrain identity based on the evolutionarily conserved expression of HOX codes and regional markers established in developmental biology. However, a direct transcriptomic comparison with fresh human embryonic hindbrain tissue was not performed due to ethical restrictions and the technical difficulty of obtaining stage-matched samples. Second, our study primarily focused on the establishment and long-term maintenance of the NSC state. While we demonstrated the general neurogenic potential and competence to respond to patterning cues, we did not establish optimized differentiation protocols to strictly enrich for specific neuronal subtypes. Developing such refined differentiation strategies will be essential for utilizing this model to dissect functional properties in a specific disease context. Finally, regarding the *in vivo* functionality, our transplantation assays demonstrated the survival and neuronal differentiation of the grafted cells; however, detailed quantification of axonal projections and functional integration into host neural circuits remains to be fully elucidated.

## Resource availability

### Lead contact

Further information and requests for resources should be directed to the lead contact, Makoto Ikeya (ikeya-g@cira.kyoto-u.ac.jp).

### Materials availability

This study did not generate new, unique reagents.

### Data and code availability

• Single-cell RNAseq data and RNAseq data generated in this study have been deposited to GEO: GSE317025 and GSE317319. We used RNAseq data of human iPSCs deposited to GEO: GSE206128. They are publicly available as of the date of publication. Other data and images that support the findings of this study are available on request from the lead contact.
• The code for generating most of the figures can be found at https://doi.org/10.5281/zenodo.18653633.
• Any additional information required to reanalyze the data reported in this paper is available from the lead contact upon request.

## Acknowledgments

We would like to express our thanks to Fabian Raudzus, Takahiro Kitahara, Edvinas Cerniauskas, Toru Kondo, Tomohisa Kato Jr., Susanna B. Mierau, and Sanjana Potlapelly for scientific discussions; to Yoichi Kosodo for material provision; and to Yoshiko Inada, Kanichiro Nakano, Yukiko Nakagawa, Nakako Shimazu, Misako Shimotsuji, and Minako Shimazoe for administrative support. We thank the CiRA Foundation for single-cell RNA sequencing; the CiRA Common Equipment Management Office for providing the research instruments, such as qPCR and confocal microscope; and Marie Obien for technical support with MEA analyses. Z.A.-A. would specifically like to express gratitude to the Otsuka Toshimi Scholarship Foundation. This work was supported by the iPS Cell Research Fund, grants-in-aid for scientific research from the Japan Society for the Promotion of Science (JSPS) (no. 16H05447), and Japan Agency for Medical Research and Development (AMED) under grant numbers JP15bm0104001 and JP23bm1323001 to M.I. This work was also supported by JST SPRING, grant number JPMJSP2110, to Z.A.-A.

## Author contributions

Conceptualization, Z.A.-A., M.N., A.M., and M.I.; methodology, Z.A.-A., D.Z., N.B.-G., M.N., T.K., A.M., and M.I.; investigation, Z.A.-A., D.Z., N.B.-G., N.E.W., M.N., T.K., and A.M.; formal analysis, Z.A.-A., M.N., and A.M.; writing – original draft, Z.A.-A.; writing – review and editing, M.N., T.K., A.M., J.T., and M.I.; supervision, N.B.-G., M.N., A.M., J.T., and M.I.; funding acquisition, Z.A.-A. and M.I.

## Declaration of interests

Z.A.-A., M.N., A.M., M.I., and Kyoto University have filed a patent application related to the findings of this study.

## Declaration of generative AI and AI-assisted technologies in the writing process

The authors declare no use of generative or assistive AI.

## STAR★Methods

### Key resources table

**Table:** 

REAGENT or RESOURCE	SOURCE	IDENTIFIER
**Antibodies**		

SOX1	Cell Signaling	4194S
SOX2	R&D systems	MAB2018
NESTIN	R&D systems	MAB1259
NANOG	R&D systems	AF1997
POU5F1	Santa Cruz Biotechnology	sc-5279
PAX6	Abcam	EPR15858
TUBB3	GeneTex	GTX85469
OLIG2	GeneTex	GTX132732
GFAP	Santa Cruz Biotechnology	sc-33673
hNCAM	Santa Cruz Biotechnology	sc-106

**Chemicals, peptides, and recombinant proteins**		

A-83-01	Tocris	2939
CHIR9902	Axon	1386
LDN193189	MedChem Express	HY-12071
Y-27632	WAKO	253–00513
iMatrix-511	Nippi	892021
Neurobasal™	ThermoFisher	21103049
DMEM/F-12	Gibco	11320–033
B27	ThermoFisher	17504044
*N*-2	ThermoFisher	17502048
GlutaMAX™	Gibco	35050
Ascorbic acid	Nacalai Tesque	03420–52
Insulin	WAKO	097–06474
BDNF	BioLegend	788902
GDNF	BioLegend	760402

**Deposited data**		

Single cell RNAseq data	This paper	GEO: GSE317025
RNAseq data	This paper	GEO: GSE317319
RNAseq datasets of human iPSCs	Kamiya et al.^18^	GEO: GSE206128

**Experimental models: Cell lines**		

1231A3 iPSC	Obtained from the Yamanaka laboratory, CiRA, Japan	https://cellbank.brc.riken.jp/cell_bank/CellInfo/?cellNo=HPS0381&lang=En
RPChiPS771-2	ReproCELL,	N/A
Ff-XT28s05-Abo_To	CiRA foundation, CiRA, Japan	https://www.cira-foundation.or.jp/e/assets/file/provision-of-ips-cells/E_Ff-XT28s05_ABo_To.pdf

**Experimental models: Organisms/strains**		

NSG mice	The Jackson Laboratory	N/A

**Oligonucleotides**		

RT-qPCR primers, see Table S1	This paper	N/A

**Software and algorithms**		

Python 3.11	https://www.python.org/downloads/	N/A
RStudio	https://posit.co/download/rstudio-desktop/	N/A
Custom code for figure generation	This paper	https://doi.org/10.5281/zenodo.18653633

### Experimental model and study participant details

#### Human iPSC cells

The human iPS cell line 1231A3 were generated from healthy donors (RIKEN BRC HPS0381; peripheral blood, female, 29 years; RRID: CVCL_LJ39). Ff-XT28s05 (HLA homozygous iPS cell line with 3rd most frequent haplotype in Japan) iPS cell line was established in FiT (Facility for iPS cell Therapy) in CiRA, Kyoto University. Both iPS cell lines were generated under written consent with the approval by the Ethics Committee at Faculty of Medicine or CiRA, Kyoto University. The RPChiPS771-2 (ReproCELL) were provided by ReproCELL.

#### NSG immunodeficient mice

NSG mice (NOD.Cg-Prkdc^scid^Il2rg^tm1Wjl^/SzJ, The Jackson Laboratory, stock no. 005557) were used in this study.

### Method details

#### iPSC culture

We used the same iPSC lines and culture method indicated in our previous report.^19^ Briefly, iPSC lines 1231A3 (obtained from the Yamanaka laboratory), RPChiPS771-2 (SgT5-2) (Stemgent), and Ff-XT28s05-Abo_To (HLAKO) (CiRA Foundation), were cultured in iMatrix-511 silk (Nippi, 892021)-coated plastic 6-well plates in StemFit AK03N (Ajinomoto) under xeno-free conditions. The cells were passaged every week by dissociating them with Accutase (Sigma–Aldrich, A6964).

#### Hb-LiNSC induction

After dissociation of iPSCs into suspension, 20,000 cells were plated in iMatrix-511-coated 12-well plastic plates in StemFit Basic03 (equivalent to the iPSC culture medium (AK03N) without bFGF), supplemented with 5 μM of A-83-01 (Tocris, 2939), 5 μM CHIR9902 (Axon, 1386), and 0.1 μM LDN193189 (MedChem Express, HY-12071) (referred to as “ACL”) along with 1 μM of Y-27632 (WAKO, 253–00513). The next day, the medium was completely changed with one without Y-27632. The AK03N medium without bFGF and with ACL was used for the maintenance of Hb-LiNSCs throughout the cell culture. Medium was changed every other day for the first 4 days then every day after day 5 of plating. Passage was carried out every week by dissociating the cells with Accutase, and Y-27632 was always added on the first day after plating. Cryopreservation was performed on dissociated cells using STEM-CELLBANKER - GMP (ZENOAQ, CB047) according to the manufacturer’s instructions.

#### Neurosphere formation and Hb-LiNSC differentiation

Hb-LiNSCs were collected as dissociated cells, and approximately 10,000 cells were seeded in each well of 96-well U-bottom low-attachment plates in NDM consisting of Neurobasal (ThermoFisher, 21103049) mixed 1:1 with DMEM/F12 (Gibco, 11320-033), supplemented with 1% B27 (ThermoFisher, 17504044), 1% *N*-2 (ThermoFisher, 17502048), 1% GlutaMAX (Gibco, 35050), 200 μM ascorbic acid (Nacalai Tesque, 03420-52), insulin 7 μg/mL (WAKO, 097–06474), BDNF 10 ng/mL (BioLegend, 788902), and GDNF 10 ng/mL (BioLegend, 760402). The cells were left to form sphere-like aggregates (neurospheres); half of the medium was changed every other day. Neurospheres were removed from the U-bottom wells and attached to the flat surface on culture plastic slides (Ibidi, IB80606) for the differentiation experiment. Neurospheres were also used for the MEA recording and for the *in vivo* experiment, the results of which are presented in Figure 5.

#### Real-time quantitative PCR analysis

RT-qPCR was performed using THUNDERBIRD Next SYBR (TOYOBO, QPX-201) and specifically designed primers (listed in Table S1) on QuantStudio 3 and QuantStudio 7 Flex Real-Time PCR Systems (Applied Biosystems), as described previously.^19^ Data from three biological replicates were analyzed to determine the relative fold change using the 2^−ΔΔCT^ method. Graphs were created using the GraphPad Prism 9 software.

#### Immunocytochemistry

Hb-LiNSCs and/or attached neurospheres were fixed with 4% paraformaldehyde and then stained with antibodies against SOX1 (Cell Signaling, 4194S), SOX2 (R&D systems, MAB2018), NESTIN (R&D systems, MAB1259), NANOG (R&D systems, AF1997), POU5F1 (Santa Cruz Biotechnology, sc-5279), PAX6 (Abcam, EPR15858), TUBB3 (GeneTex, GTX85469), OLIG2 (GeneTex, GTX132732), or GFAP (Santa Cruz Biotechnology, sc-33673), and with DAPI for the nuclei. The stained cells were visualized using a Keyence microscope (BZ-X710 or BZ-X810) and an Olympus 3000 confocal microscope system.

#### RNA sequencing

For the bulk RNA-seq, cell lysis and RNA extraction and sequencing were performed as described previously.^19^ For analyzing the count data, we used the DESeq2 method for normalization and for differential gene expression analysis.^20^ To generate the plots, we used “EnhancedVolcano,” “ComplexHeatmap,” “ggVennDiagram,” “ggplot2,” and other standard RStudio packages. For pathway enrichment, we used Enrichr.^21^ As for scRNA-seq, we processed 5000 cells from each sample and prepared the sequencing library using the Chromium Next GEM Single Cell 3′ Reagent Kits v3.1 from 10x Genomics, according to the standard procedures recommended by the manufacturer (Dual Index). After cDNA synthesis, size (TapeStation) and concentration (Qubit) measurements were performed. The size (TapeStation) and concentration (qPCR method) were performed after library preparation. The processing, filtration, analysis, and plotting of data were done in Python using Scanpy.^22^

#### Multielectrode array recording

High-density multiple electrode arrays (HD-MEAs) from MaxWell Biosystems were used for detecting neuronal firing and network activity in the neurospheres. One-week-old neurospheres were plated directly onto the iMatrix-511-coated electrode after formation in suspension. Recording was done after six weeks on a MaxOne HD-MEA system. The same electrodes were recorded again after 1 h of 1 μM TTX treatment. The data were analyzed using the MaxLab Live software.

#### *In vivo* neurosphere implantation

Approximately 4-week-old mice were used for the transplantation of one-week-old neurospheres. The neurospheres (about five spheres) were collected with a Hamilton syringe using a stereotactic device and then delivered into the hindbrain region of the mouse brain after drilling the cranium, referring to the coordinates from the mouse brain atlas. After 8 weeks of transplantation, mice were sacrificed according to the ethical guidelines, and then perfusion fixation was performed. Whole brain samples were collected and fixed with 4% paraformaldehyde and then incubated in 30% sucrose for 24 h. The samples were then embedded into a freezing compound for cryosectioning. Thirty-micrometer-thick sections were cut in the sagittal plane of the transplanted hemispheres.

### Quantification and statistical analysis

Unpaired parametric Welch’s *t* test was performed using the GraphPad Prism 9 software. Significance levels were defined as n.s. > 0.05, ∗*p* ≤ 0.05, ∗∗*p* ≤ 0.01, ∗∗∗*p* ≤ 0.001, and ∗∗∗∗*p* ≤ 0.0001. Details are described in the figure legends.

## Supplemental information

Document S1. Figures S1–S4 and Table S1

Document S2. Article plus supplemental information

## References

[1] Metzis V., Steinhauser S., Pakanavicius E., Gouti M., Stamataki D., Ivanovitch K., Watson T., Rayon T., Mousavy Gharavy S.N., Lovell-Badge R. (2018). Nervous System Regionalization Entails Axial Allocation before Neural Differentiation. Cell.

[2] Altman J., Das G.D. (1965). AUTORADIOGRAPHIC AND HISTOLOGICAL EVIDENCE OF POSTNATAL HIPPOCAMPAL NEUROGENESIS IN RATS. J. Comp. Neurol..

[3] Tailor J., Kittappa R., Leto K., Gates M., Borel M., Paulsen O., Spitzer S., Karadottir R.T., Rossi F., Falk A., Smith A. (2013). Stem Cells Expanded from the Human Embryonic Hindbrain Stably Retain Regional Specification and High Neurogenic Potency. J. Neurosci..

[4] Takahashi K., Yamanaka S. (2006). Induction of pluripotent stem cells from mouse embryonic and adult fibroblast cultures by defined factors. Cell.

[5] Fukusumi H., Shofuda T., Bamba Y., Yamamoto A., Kanematsu D., Handa Y., Okita K., Nakamura M., Yamanaka S., Okano H., Kanemura Y. (2016). Establishment of Human Neural Progenitor Cells from Human Induced Pluripotent Stem Cells with Diverse Tissue Origins. Stem Cells Int..

[6] Rifes P., Isaksson M., Rathore G.S., Aldrin-Kirk P., Møller O.K., Barzaghi G., Lee J., Egerod K.L., Rausch D.M., Parmar M. (2020). Modeling neural tube development by differentiation of human embryonic stem cells in a microfluidic WNT gradient. Nat. Biotechnol..

[7] Philippidou P., Dasen J.S. (2013). Hox genes: choreographers in neural development, architects of circuit organization. Neuron.

[8] Joyner A.L., Liu A., Millet S. (2000). Otx2, Gbx2 and Fgf8 interact to position and maintain a mid-hindbrain organizer. Curr. Opin. Cell Biol..

[9] Lagutin O.V., Zhu C.C., Kobayashi D., Topczewski J., Shimamura K., Puelles L., Russell H.R.C., McKinnon P.J., Solnica-Krezel L., Oliver G. (2003). Six3 repression of Wnt signaling in the anterior neuroectoderm is essential for vertebrate forebrain development. Genes Dev..

[10] Lu J., Zhong X., Liu H., Hao L., Huang C.T.L., Sherafat M.A., Jones J., Ayala M., Li L., Zhang S.C. (2016). Generation of serotonin neurons from human pluripotent stem cells. Nat. Biotechnol..

[11] Bane V., Lehane M., Dikshit M., O'Riordan A., Furey A. (2014). Tetrodotoxin: Chemistry, Toxicity, Source, Distribution and Detection. Toxins.

[12] Aziz-Bose R., Monje M. (2019). Diffuse intrinsic pontine glioma: molecular landscape and emerging therapeutic targets. Curr. Opin. Oncol..

[13] Sit T.P.H., Feord R.C., Dunn A.W.E., Chabros J., Oluigbo D., Smith H.H., Burn L., Chang E., Boschi A., Yuan Y. (2024). MEA-NAP: A flexible network analysis pipeline for neuronal 2D and 3D organoid multielectrode recordings. Cell Rep. Methods.

[14] Kagan B.J., Kitchen A.C., Tran N.T., Habibollahi F., Khajehnejad M., Parker B.J., Bhat A., Rollo B., Razi A., Friston K.J. (2022). In vitro neurons learn and exhibit sentience when embodied in a simulated game-world. Neuron.

[15] Beaubois R., Cheslet J., Duenki T., De Venuto G., Carè M., Khoyratee F., Chiappalone M., Branchereau P., Ikeuchi Y., Levi T. (2024). BiœmuS: A new tool for neurological disorders studies through real-time emulation and hybridization using biomimetic Spiking Neural Network. Nat. Commun..

[16] Cai H., Ao Z., Tian C., Wu Z., Liu H., Tchieu J., Gu M., Mackie K., Guo F. (2023). Brain organoid reservoir computing for artificial intelligence. Nat. Electron..

[17] Wu Y., Chen X., Xi G., Zhou X., Pan S., Ying Q.L. (2019). Long-term self-renewal of naive neural stem cells in a defined condition. Biochim. Biophys. Acta. Mol. Cell Res..

[18] Kamiya D., Takenaka-Ninagawa N., Motoike S., Kajiya M., Akaboshi T., Zhao C., Shibata M., Senda S., Toyooka Y., Sakurai H. (2022). Induction of functional xeno-free MSCs from human iPSCs via a neural crest cell lineage. NPJ Regen. Med..

[19] Al-Akashi Z., Zujur D., Kamiya D., Kato T., Kondo T., Ikeya M. (2023). Selective vulnerability of human-induced pluripotent stem cells to dihydroorotate dehydrogenase inhibition during mesenchymal stem/stromal cell purification. Front. Cell Dev. Biol..

[20] Love M.I., Huber W., Anders S. (2014). Moderated estimation of fold change and dispersion for RNA-seq data with DESeq2. Genome Biol..

[21] Kuleshov M.V., Jones M.R., Rouillard A.D., Fernandez N.F., Duan Q., Wang Z., Koplev S., Jenkins S.L., Jagodnik K.M., Lachmann A. (2016). Enrichr: a comprehensive gene set enrichment analysis web server 2016 update. Nucleic Acids Res..

[22] Wolf F.A., Angerer P., Theis F.J. (2018). SCANPY: large-scale single-cell gene expression data analysis. Genome Biol..

